# A novel learner driver first aid eLearning program: a mixed-method pre-post pilot test and evaluation

**DOI:** 10.1186/s12873-024-01036-4

**Published:** 2024-07-29

**Authors:** Olivia Miller, Sharon Newnam

**Affiliations:** School of Psychology and Counselling, Faculty of Health, Queensland University of Technology, O Block, Wing B, Level 5, Brisbane, Australia

**Keywords:** First aid, Prehospital care, Learner drivers, eLearning, Road safety

## Abstract

**Background:**

Approximately 1.35 million people worldwide are killed in road accidents every year. Mandatory first aid training for learner drivers has been introduced in some European countries but no such requirements are in effect in Australia. The current study aimed to pilot and evaluate a first aid eLearning program for Australian learner drivers undertaking their mandated supervised driving hours.

**Methods:**

A total of 103 participants (*M* age = 20.57; 52.4% female, 96% completion rate) responded to an online survey immediately before and two weeks after completing the Learner Driver First Aid program. Participants completed measures of first aid self-efficacy, first aid knowledge, and attitudes towards first aid, and provided qualitative feedback on the program. Paired samples t-tests and Mann-Whitney U tests assessed improvements in first aid self-efficacy, knowledge, and attitudes, and qualitative feedback were analysed thematically.

**Results:**

Participants showed significant pre-post program improvements in first aid self-efficacy (*p* < .001) and first aid knowledge (*p* < .001); however, there were no significant changes in attitudes towards first aid (*p* = .028). Self-efficacy and knowledge improvements were significantly greater for those without prior first aid training (*p* < .001). Participants rated the usability of the online program favourably and most (93.2%) were satisfied or extremely satisfied with the program. Qualitative feedback suggested participants found the program to be accessible and interactive but noted concerns about the transfer of skills to the real-world context.

**Conclusions:**

The findings provide support for the efficacy of online first aid training for Australian learner drivers. However, further improvements to the eLearning program based on participant feedback should be considered. This study recommends the Learner Driver First Aid program be refined and rolled out to the Australian public.

**Supplementary Information:**

The online version contains supplementary material available at 10.1186/s12873-024-01036-4.

## Background

Road accidents are common and can result in significant harm. According to the Australian Institute of Health and Welfare, [1] in 2020-21 there were 61,500 injuries and 1400 deaths due to transport accidents in Australia. Road crash injuries are similarly high worldwide and 1.35 million people die each year because of road incidents [2]. Of concern, adolescents are over-represented in transport related mortality rates despite making up a small proportion of road users [3]. Initiatives aimed at reducing transport-related injury and death tend to focus on changing driver behaviours without recognising the role of bystanders.

Bystanders play an important role in responding to road incidents prior to the arrival of emergency services [4]. For example, medical intervention by a first aid trained person in the immediate period following a road crash can prevent death and lessen the impact of injuries, [5, 6] and first aid training can increase bystander willingness and confidence to intervene [7, 8]. Unfortunately, however, many people lack the skills and training to adequately respond in emergencies [7, 9, 10].

Subsequently, the World Health Organization emphasised the importance of training drivers in first aid [11]. First aid training can improve road user first aid knowledge, though few studies have focused on learner drivers specifically [12]. In Australia people can obtain a learner class driving licence from age 16–17 upwards by passing a written exam. Before they can progress to a provisional license and drive unsupervised, they must complete 50–120 h (depending on the jurisdiction) of supervised driving. They remain classed as a learner driver while completing their supervised driving hours. Preliminary evidence suggests that first aid training can significantly improve learner driver first aid knowledge and self-efficacy (SE) or their belief in their ability to provide first aid. For example, a Danish evaluation of face-to-face training found that it significantly improved learner drivers’ first aid SE and knowledge [13]. However, participant knowledge was tested immediately before and after training meaning knowledge retention was not assessed. Similarly, a Czech evaluation found that learner drivers who engaged in face-to-face training had significantly higher first aid knowledge compared to those who engaged in the traditional learner driver first aid training (watching videos) [14]. A follow-up study showed that video training was significantly better than none at all [15]. The existing evidence suggests that first aid training for learner drivers can improve first aid knowledge and potentially reduce road harm.

While there is preliminary support for the effectiveness of learner driver first aid training, [13–15] it is unclear if online training has the same benefits. For example, a comparison of interactive face-to-face first aid training, lecture-based training, and video instruction for Chinese teachers found that face-to-face training resulted in significantly higher first aid knowledge compared to other modalities [16]. Similarly, lecture based neonatal cardiopulmonary resuscitation training supplemented with a demonstrative video resulted in significantly higher knowledge and skill improvements in comparison to lecture based training supplemented with additional lecture slides (rather than a video demonstration) [17]. A meta-analysis on the effectiveness of different learning modalities for health professionals found that there were no significant differences in knowledge acquisition between digital problem-based learning and traditional face-to-face problem-based learning; however, digital problem-based learning resulted in significantly higher skill acquisition in comparison to traditional face-to-face problem-based learning [18]. Another meta-analysis found that students in blended learning conditions performed better than those receiving traditional face-to-face instruction [19]. Both online and face-to-face modalities tend to receive similar satisfaction ratings though online learning is appreciated for its flexibility [20, 21].

Despite calls for learner driver first aid training and initial evidence of its effectiveness, [11, 13–15] no Australian jurisdiction mandates or delivers learner driver first aid training. To address this, St John Ambulance Australia developed the Learner Driver First Aid program (https://driverfirstaid.org.au), a free 30-minute online course teaching first aid skills to be applied at the scene of a transport incident before emergency services arrive. The program covers infection control, basic life support, and first aid for wounds and bleeding. It is interactive and includes pictorial demonstrations, voice over explanations, clickable prompts, revision questions, and personalized completion certificates.

The current study aimed to pilot and evaluate the Learner Driver First Aid program in Australia. It was considered a pilot study because it was a small-scale evaluation of the intervention to assess the feasibility of further large-scale evaluation (e.g., longitudinal randomised controlled trial). Program evaluation included assessment of first aid knowledge, SE, attitudes towards first aid, and program satisfaction. It was hypothesised that participants would show pre- to post-program improvements in first aid knowledge, SE, and attitudes towards first aid and would be largely satisfied with the program.

## Methods

### Participants

Using a generic sample size calculation for paired sample t-tests with a medium effect size (*d* = 0.50), 90% power, and a Bonferroni corrected alpha level of 0.004, the required sample size was 74. A sample size calculation for an independent sample t-test with a medium effect size (*d* = 0.50) and 80–90% power, indicated the required sample size was 102–140 (calculations conducted using G*Power Version 3.1) [22]. Therefore, the current study aimed to recruit 100 participants which was achieved as 103 Australian residents with a current learner class driving license participated. Approximately half were women (52.4%), drove in the city (49.5%), and had prior first aid training (51.5%). They ranged between 16 and 48 years of age (*M* = 20.57, *SD* = 5.02), had obtained their learner license between 1 and 84 months previously (*M* = 13.59, *SD* = 17.41), and drove between 0 and 40 supervised hours per week (*M* = 8.21; *SD* = 7.31). Participants were recruited through social media advertisements, the university Participate in Research webpage, and/ or driving schools. Participants were offered a $50 e-voucher following completion of the study.

### Learner driver first aid program

The Learner Driver First Aid program was developed by a team of first aid experts from St John Ambulance Australia to align with the Australian and New Zealand Committee on Resuscitation (ANZCOR) first aid guidelines [23]. The program was developed with a specific focus on the roadside context by focusing on safety factors specific to a road accident (e.g., making sure it is safe to approach a vehicle, whether or not to remove someone from their vehicle) and first aid skills that may be needed at the scene of a road incident (e.g., internal bleeding). It is delivered online, takes approximately 30 min to complete, and is interactive including engaging graphics, clickable prompts, and revision questions assessing knowledge on the first aid skills covered by the program. As seen in Table 1, the program consists of three overarching segments, each with their own sub-segments. Each overarching segment finishes with revision questions that learners must get correct before progressing to the next segment.

**Table 1:** Segments of the learner driver first aid program

Program Segment	Description
1. Introduction: What is First Aid? a) Duty of care b) Consent c) Debriefing d) Infection control	Introduction to first aid including information on duty of care, consent, the importance of debriefing, and infection control.
2. Basic Life Support (DRSABCD) a) What is DRSABCD? b) D – Danger c) R – Response d) S – Send for help e) A – Airway f) B – Breathing g) C – CPR h) D – Defibrillation i) Recovery position	Instruction on basic life support practices including how to put someone in the recovery position and how to enact the basic life support action plan (DRSABCD).
3. Wounds and bleeding a) External bleeding b) Management of external bleeding c) Embedded object d) Internal bleeding e) Management of internal bleeding	Instruction on how to treat wounds and bleeding that may occur at a roadside incident. This segment covers first aid for external bleeding, embedded objects, and internal bleeding.

### Measures

#### Time 1 (Pre-Intervention) and Time 2 (Post-Intervention): Program Effectiveness

A 5-item SE scale was developed for the study using Bandura’s recommendations [24]. Items were developed to assess participants’ confidence to perform the first aid skills taught in the program including (a) providing first aid, (b) providing basic life support, (c) placing someone in the recovery position, (d) managing external injuries, and (e) managing internal bleeding. Participants rated their confidence to perform these skills on a Likert scale ranging from 0 (*cannot do at all*) to 100 (*highly certain can do*). Scores were averaged and higher scores indicated higher SE. The scale had adequate internal consistency (Time 1 α = 0.95; Time 2 α = 0.90), [25] and test-retest reliability (*r* = .72, *p* < .001) [26].

Positive attitudes towards first aid were measured using a previously developed 4-item scale, [27] with items adapted to refer to the roadside context (see items in Table 2 below). Participants rated their degree of agreement with each item on a 5-point Likert scale ranging from 0 (*strongly disagree*) to 5 (*strongly agree*). In the current study, the scale demonstrated adequate test-retest reliability (*r* = .63, *p* < .001), [26] but less than adequate internal consistency (Time 1 α = 0.52; Time 2 α = 0.45) [25].

A 10-item multiple choice quiz was created for the study to assess first aid knowledge (see supplementary material). Previously developed first aid knowledge assessments could not be used because they focused on practical hands-on skill demonstrations and/ or because they assessed first aid knowledge not taught in the program. Items were based on the program content, prior research, and the ANZCOR first aid guidelines, [23, 27, 28] and were developed in consultation with first aid experts from St John Ambulance Australia. Only questions relevant to program content were included and questions were modified to ensure they did not replicate revision questions asked within the program. Responses were rated as correct (1 point) or incorrect (0 points) and points were summed to get an overall knowledge score ranging from 0 to 10. However, the scale demonstrated less than adequate test-retest reliability (*r* = .30, *p* = .003), [26] and internal consistency (Time 1 α = 0.44; Time 2 α = 0.24) [25]. Note that poor test-retest reliability is not of concern given that knowledge was expected to change over time because of the intervention.

#### Time 2 (Post-Intervention) only: Program experiences

The usability of the first aid program was assessed using the 10-item System Usability Scale (SUS) [29]. Participants rated their experience of using the program on a 5-point Likert scale ranging from 1 (*strongly disagree*) to 5 (*strongly agree*). Total scores ranged from 0 to 100 with higher scores indicating better system usability [29]. The SUS is a valid and reliable measure for a variety of systems, [30, 31] as confirmed by the current study (α = 0.81) [25].

Eight items developed for the current study assessed whether the learning objectives were met. Items were developed in consultation with St John Ambulance Australia based on the learning objectives of the program which were written to align with ANZCOR guidelines [23]. Namely, the program aimed to teach the following first aid skills: infection control, basic life support, management of external bleeding, and management of internal bleeding. Items evaluated how well participants felt the program explored (4 items) and instructed (4 items) on each of these skills. Participants rated their agreement with each statement on a 5-point Likert scale ranging from 1 (*strongly disagree*) to 5 (*strongly agree*). This scale demonstrated adequate internal consistency (α = 0.89) [25].

Participants rated their satisfaction with the design and delivery of the program using three previously developed items, [32] adapted slightly to suit the online learning environment. Participants rated their agreement with the following items: “the content of the program was relevant”, “the online delivery was effective and accessible” and “the resource materials were useful” using a 5-point Likert scale ranging from 1 (*strongly disagree*) to 5 (*strongly agree*). Higher scores indicated higher satisfaction, and the current study confirmed adequate reliability (α = 0.78) [25].

Similarly, participants rated their satisfaction with the program and its content (infection control, basic life support, external bleeding, internal bleeding) over four items using a 5-point Likert scale ranging from 1 (*not at all satisfied*) to 5 (*extremely satisfied*). This scale was developed for the current study so that items could be tailored to the program content, and response options were adopted from validated and reliable satisfaction measures [33, 34]. Higher scores indicated higher satisfaction. This scale had adequate internal consistency (α = 0.81) [25].

#### Qualitative measures

The survey closed with three open-ended questions asking participants to briefly (a) describe what they liked about the program, (b) describe what they did not like about the program, and (c) provide suggestions for improving the program.

### Procedure

All procedures were performed in compliance with relevant laws and guidelines, and approval was obtained by the university human research ethics committee (approval number 6747). Participants clicked on the survey link directly from recruitment materials, provided informed consent, created a unique participant code so that their data could be linked over time, and completed the first survey. The survey closed with instructions to complete the Learner Driver First Aid program and then email their personalised completion certificate to the researcher. Two weeks after receipt of participants’ completion certificates, participants were emailed with the link to complete the second survey. After confirming completion of all study components (both surveys and the Learner Driver First Aid program), participants were emailed a $50 e-voucher and then identifiable information was destroyed. The completion rate was 96% with only 4% attrition.

### Analyses

A series of paired samples t-tests assessed whether participants showed pre- to post-program improvements in their first aid SE, knowledge, and attitudes towards first aid. A series of Mann-Whitney U tests assessed whether demographic characteristics impacted changes to first aid SE and knowledge. Descriptive data (*M*, frequencies) evaluated the program usability, design, delivery, and participant satisfaction. Qualitative analysis was underpinned by a realist framework and conducted thematically using template analysis, [35] with an inductive and semantic approach to coding. Template analysis began with data familiarisation including several readings of participant responses. Preliminary coding then commenced on a subset of the data (*n* = 10) so that an initial coding template could be developed. This initial coding template was based off the qualitative questions and included satisfaction, challenges, and changes, and sub-codes were developed iteratively in accordance with participant responses. The initial coding template was then applied to the remaining data and refined as necessary (see final template in Table 4 below). For example, initial satisfaction sub-codes of online delivery, structure, and completion time were combined to represent program accessibility. One researcher (OM) conducted the qualitative analysis and engaged in reflexivity through frequent discussion with the other author (SN) who endorsed the analysis.

## Results

### Program Effectiveness

**Fig. 1:**
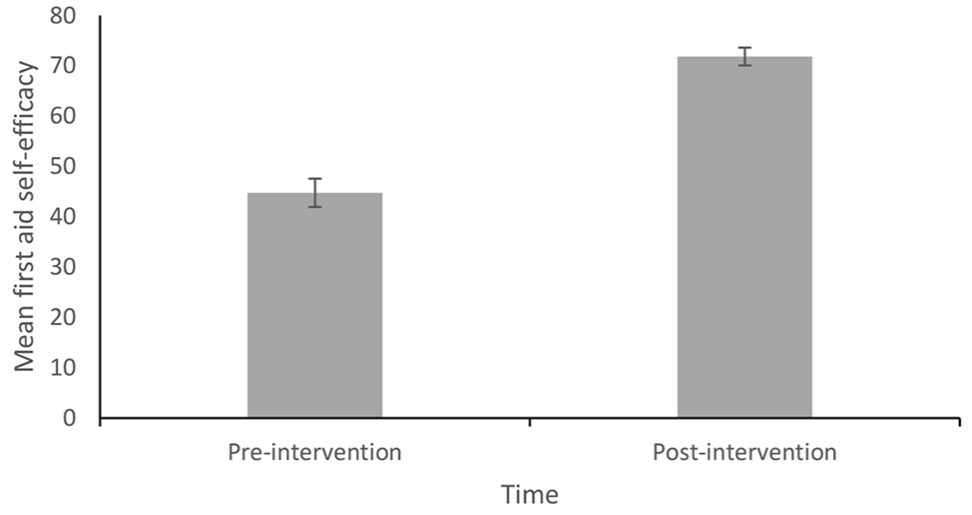
Average Pre- and Post-Intervention Total First Aid Self-Efficacy Scores. Note: Error bars represent standard error

**Fig. 2:**
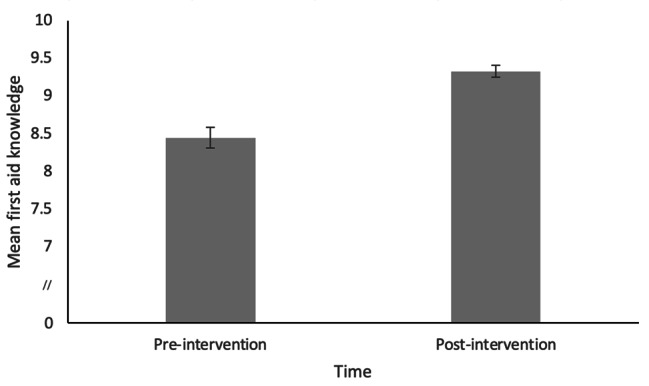
Average Pre- and Post-Intervention First Aid Knowledge Scores. Note: Error bars represent standard error

**Fig. 3:**
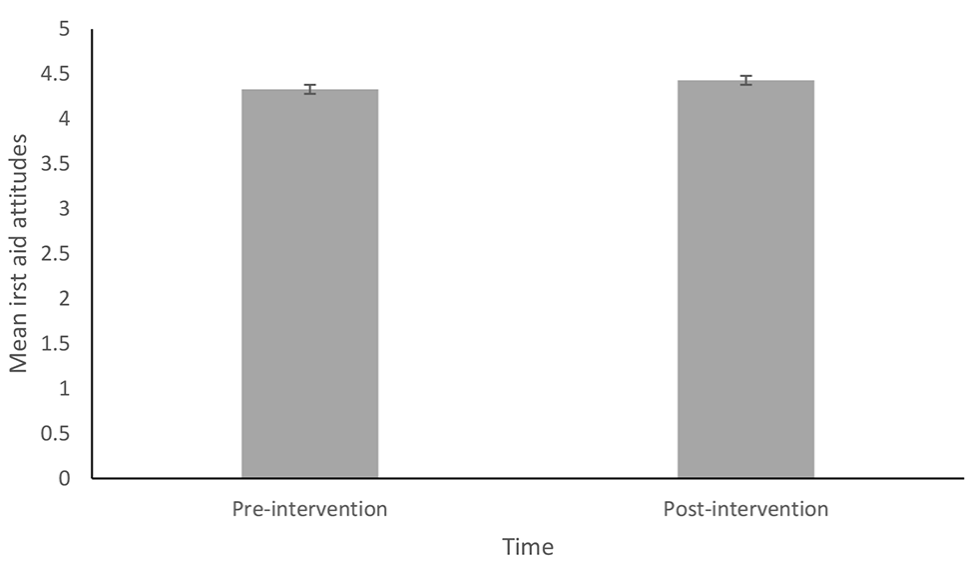
Average Pre- and Post-Intervention Attitudes Towards First Aid Scores. Note: Error bars represent standard error

Histograms of difference scores showed that the distribution of differences for each variable were roughly normally distributed. The data were assessed for outliers using histograms and boxplots which revealed some outliers. Analyses were performed with and without outliers, and subtle differences in results were obtained (attitude items became non-significant). Therefore, outliers were excluded from all subsequent analyses. As shown in Table 2; Figs. 1, 2 and 3, paired samples t-tests showed significant improvements in total first aid SE and first aid knowledge, but not in attitudes towards first aid.

**Table 2:** Paired sample t-tests of first aid self-efficacy, first aid attitudes, and first aid knowledge

Variable/ item	T1 M	T2 M	t	df	95% CI	*p*	d
Total first aid SE.	44.76	71.83	-13.80	102	[-30.96, -23.18]	< 0.001^*^	0.95
SE1: Provide first aid at the scene of a motor vehicle accident.	44.71	73.14	-11.87	101	[-33.19, -23.69]	< 0.001^*^	0.89
SE2: Follow the DRSABCD action plan (i.e., provide basic life support).	47.28	78.45	-11.74	102	[-36.43, -25.90]	< 0.001^*^	0.96
SE3: Safely place someone in the recovery position.	53.76	78.32	-9.86	100	[-29.50, -19.61]	< 0.001^*^	0.81
SE4: Safely manage external injuries.	48.43	73.53	-11.33	101	[-29.49, -20.70]	< 0.001^*^	0.84
SE5: Safely manage internal injuries.	30.87	57.96	-11.54	102	[-31.75, -22.43]	< 0.001^*^	0.85
Total first aid attitudes	4.33	4.43	-2.24	102	[-0.20, -0.01]	0.028	0.19
A1: Giving first aid at a roadside incident scene increases the victims’ survival rate.	4.66	4.74	-1.21	95	[-0.22, 0.05]	0.230	0.18
A2: It is important to learn about first aid to be able to help others at a roadside incident scene.	4.62	4.69	-1.19	97	[-0.19, 0.05]	0.239	0.12
A3: Giving first aid is NOT good at a roadside incident scene (R).	0.38	0.23	2.47	90	[0.03, 0.28]	0.16	0.24
A4: Bystanders have a responsibility to give first aid at a roadside incident scene.	3.81	3.98	-1.40	97	[-0.42, 0.07]	0.165	0.17
Total knowledge.	8.45	9.33	-6.37	97	[-1.15, -0.60]	< 0.001^*^	0.64

Note. T1 = time 1 (pre-intervention). T2 = time 2 (post-intervention). SE = self-efficacy item. A = Attitudes item. R = reverse scored. ^*^ survived Bonferroni correction (0.05/12 = 0.004)

### Program effectiveness by demographics

Total first aid SE and knowledge scores were transformed into change scores (Time 2-Time 1) where positive scores indicated SE or knowledge improvements and negative scores represented SE or knowledge reductions. As SE and knowledge change scores were not normally distributed the non-parametric Mann-Whitney U test was conducted. As seen in Table 3, there were no significant differences in SE or knowledge change scores based on gender (male vs. female) or primary driving location (city vs. outside city). However, there were significant differences based on prior first aid training. Those without prior first aid training had significantly greater first aid SE (*Mdn* = 38.00) and knowledge (*Mdn* = 1.00) improvements compared to participants with prior first aid training (*Mdn*_SE change_ = 10.00, *Mdn*_knowledge change_ = 0.00).

**Table 3:** Differences in knowledge and SE change scores by demographics

	SE change					Knowledge change			
Demographic variable	*U*	*z*	*p*	*r*		*U*	*z*	*p*	*r*
Gender	1357.00	0.80	0.426	0.08		1235.00	0.96	0.340	0.10
Primary driving location	1538.00	1.40	0.162	0.14		1266.50	0.51	0.613	0.05
Prior first aid training	2037.50	5.47	< 0.001^*^	0.55		1573.00	3.55	< 0.001^*^	0.36

Note. ^*^ survived Bonferroni correction

### Program experiences

The usability of the program was rated highly (*M*_SUS_ = 85.39, *SD* = 13.67). Most participants agreed or strongly agreed the content of the program was relevant (97.1%), the online delivery was effective and accessible (92.2%), the resource materials were useful (92.2%), and the program effectively explored infection control (67%), basic life support (93.2%), first aid for external bleeding (89.3%), and first aid for internal bleeding (70.9%). Similarly, most agreed or strongly agreed the program provided relevant instruction on infection control (68.9%), basic life support (92.2%), first aid for external bleeding (85.5%), and first aid for internal bleeding (70%). Most participants were satisfied or extremely satisfied with the program as a whole (93.2%), as well as the infection control (70.8%), basic life support (91.2%), external bleeding (85.5%), and internal bleeding (68%) components.

### Qualitative data

Most participants (97%) responded to at least one qualitative question (question 1, *n* = 100; question 2, *n* = 62; question 3, *n* = 44). As seen in Table 4, template analysis of this data generated three key themes including program satisfaction, program challenges, and suggestions for improvement. Briefly, participants were largely satisfied with the program due to ease of access, interactive delivery, and perceived improvements in first aid knowledge; experienced technical and skill transfer challenges; and suggested improvements like refresher training, downloadable resources, and further instruction, particularly on infection control (*n* = 7) and internal bleeding (*n* = 11).

**Table 4:** Key themes and sub-themes relating to participant experiences of the learner driver first aid program

Key theme	Sub-themes	Qualitative comments
Program Satisfaction	Accessibility	“easy to follow/ understand” (Participants 9, 22, 85) “being online was good!” (Participant 51) “it was structured well” (Participant 20)
	Interactive delivery	“I liked that there were little activities because it makes it easier to remember the information” (Participant 65)
	Knowledge improvements	“I learned a lot and I am grateful for this program” (Participant 49) “it improved my knowledge of first aid” (Participant 102)
Program Challenges	Technical difficulties	“audio sometimes went in and out on the phone” (Participant 33) “troubles with background colours on my phone” (Participant 47)
	Skill transfer	“I did not feel like there was enough information available for me to actually put these steps into use” (Participant 20) “I know the information about what do and what not to do but I wouldn’t be able to do it in practice” (Participant 31) “the experience would not translate to real life in my opinion as you don’t have a very hands-on approach while using an online website” (Participant 69)
Suggestions for Improvement	Refresher training	“It’d be great if there was a follow up refresher email” (Participant 60)
	Downloadable resources	“I would like a little handbook that I could print and put in my car” (Participant 46)
	Additional information and video demonstrations	“some videos outlining step-by-step how to do some of the things being taught would be helpful” (Participant 8) “I believe that it needs to go more in depth with diagrams and pictures explaining and demonstrating” (Participant 57)

## Discussion

This study aimed to deliver and evaluate the Learner Driver First Aid eLearning program in Australia. The program significantly improved learner drivers’ first aid SE and knowledge but not attitudes towards first aid, and participants without prior first aid training showed the greatest improvements. Additionally, most participants were satisfied or extremely satisfied with the program. These findings provide evidence for the efficacy of online first aid training for learner drivers and opportunities for improvement.

Similar to the current findings, previous evaluations of European learner driver first aid programs showed improvements in learner driver first aid knowledge and SE post intervention or in comparison to controls [13–15]. However, these previous evaluations typically measured knowledge or SE immediately after training, and training was delivered face-to-face rather than online. Therefore, the current study provides evidence that knowledge can be retained for up to two weeks and that online delivery can be an effective method of knowledge dissemination. However, further research is required to determine whether first aid knowledge and SE can translate into roadside first aid skills.

Contrary to previous research, [36] the current study did not find significant post-program improvements in attitudes towards first aid. However, there was evidence of a ceiling effect as participants had high attitude scores at baseline making it difficult to detect improvements at follow-up. The low internal consistency of the attitude measure may also have contributed to this non-significant finding.

This study identified opportunities for program improvement and rollout. Despite being satisfied with the online delivery, participants were concerned about skill transfer from the online environment. This is consistent with previous research which has shown the ability to learn and retain first aid skills is impacted by learning environment/ mode among other factors [16, 17, 37, 38]. To assist skill transfer, participants suggested including video demonstrations of first aid skills as opposed to pictorial demonstrations only which is supported by prior research [17]. As first aid knowledge and SE improvements were significantly greater for those without prior first aid training, the Learner Driver First Aid program shows promise as an introductory course for beginner first aiders. However, those with prior first aid training also showed significant improvements suggesting it may also be suitable as a refresher course.

Despite collecting both quantitative and qualitative data, evaluating the program over a 2-week period, having sufficient statistical power, and a relatively even gender split, the current study did have limitations that must be acknowledged. First, the findings cannot be solely attributed to the intervention because (a) there was no control group and (b) the knowledge measure lacked reliability. Similarly, a learning effect cannot be ruled out because participants completed the same survey measures at baseline and follow-up. However, this was minimised by testing participants 2-weeks after program completion rather than immediately after as done in prior evaluations, [13] and by blinding participants to the fact they would be completing the same measures. The recruitment strategy prevented confirmation of eligibility (e.g., learner status) and relied on participant honesty which may have been influenced by the incentive as evidenced by the low attrition rate. Finally, the effect of the program was not evaluated over time (e.g., 3-, 6-, 12-months), meaning long-term knowledge retention beyond 2-weeks could not be assessed nor could the potential real-world impacts in terms of learner drivers effectively intervening at roadside accidents.

## Conclusions

The Learner Driver First Aid program is the first of its kind in Australia and this pilot evaluation provides evidence for its effectiveness in significantly improving learner driver first aid SE and first aid knowledge. It is suggested the program be further refined and rolled out on a larger scale to the Australian public. Future research should use more rigorous designs to further explore the effect of first aid training on learner driver first aid knowledge, skills, and willingness to intervene at roadside accidents, and subsequently explore whether first aid training for learner drivers can reduce road accident mortality rates.

### Electronic supplementary material

Below is the link to the electronic supplementary material.

Supplementary Material 1

## Data Availability

The data that support the findings of this study are available from Queensland University of Technology, but restrictions apply to the availability of these data, and so are not publicly available. The data are, however, available from the authors upon reasonable request and with the permission of Australian Capital Territory (funders).

## Abbreviations

ANZCOR: Australian and New Zealand Committee on Resuscitation
SE: Self-Efficacy
