# Supplementary material for: A novel learner driver first aid eLearning program: a mixed-method pre-post pilot test and evaluation
Source: BMC Emerg Med. 2024 Jul 29;24:137. doi: 10.1186/s12873-024-01036-4 (PMC11287832; doi:10.1186/s12873-024-01036-4)
Supplement: Supplementary file 1 — Supplementary Material 1 [file 12873_2024_1036_MOESM1_ESM.docx]

**Supplementary Material**

**Supplementary Material A.**

First aid knowledge items (correct answers bolded)

1. What number should you call when you witness a road traffic crash?
   1. 000
   2. 112
   3. **Either of the above numbers**
2. How can you assess whether a person is unconscious?
   1. Ask the injured person what their name is
   2. Ask the injured person to squeeze your hand
   3. **Both of the above**
3. How can you assess if someone is breathing?
   1. Look for movement of the abdomen or chest
   2. Listen for air escaping from the nose or mouth
   3. Feel for the movement of air at the mouth and nose
   4. **All of the above**
4. You should provide CPR when:
   1. A person is unconscious but breathing normally
   2. **A person is unconscious, but their breathing is shallow and irregular**
   3. A person is conscious but has not consented to CPR
5. When performing CPR, what is the ratio of compressions to breaths?
   1. 10 compressions: 1 breath
   2. 20 compressions: 2 breaths
   3. **30 compressions: 2 breaths**
6. In what situation should you physically move an injured person at the scene of a road traffic accident?
   1. **The injured person is in a hazardous environment (e.g., on the road, in the way of oncoming traffic)**
   2. The injured person is in a car but can be managed safely there
   3. The injured person is conscious and able to safely move themselves
7. You do not need to provide first aid if you may be in danger when doing so.
   1. **True**
   2. False
8. When attending a roadside accident scene and applying first aid, it is recommended that you follow the DRSABCD action plan. What does this acronym stand for?
   1. Danger, Rest, Send for help, Airway, Breathing, Care, Defibrillation
   2. **Danger, Response, Send for help, Airway, Breathing, CPR, Defibrillation**
   3. Defibrillation, Response, Send for help, Airway, Breathing, CPR, Danger
9. If the injured person has an object embedded in a wound, you should remove it.
   1. True
   2. **False**
10. If an injured person bleeds through a bandage you should:
    1. Remove it an apply another one
    2. Remove it and apply pressure with your hands
    3. **Leave it on, and apply another one**
